# Low Serum Vitamin B12 Levels Are Associated with Adverse Lipid Profiles in Apparently Healthy Young Saudi Women

**DOI:** 10.3390/nu12082395

**Published:** 2020-08-10

**Authors:** Sara Al-Musharaf, Ghadeer S. Aljuraiban, Syed Danish Hussain, Abdullah M. Alnaami, Ponnusamy Saravanan, Nasser Al-Daghri

**Affiliations:** 1 Department of Community Health Sciences, College of Applied Medical Sciences, King Saud University, Riyadh 11451, Saudi Arabia; galjuraiban@ksu.edu.sa; 2 Chair for Biomarkers of Chronic Diseases, Riyadh Biochemistry Department, College of Science, King Saud University, Riyadh 11451, Saudi Arabia; danishhussain121@gmail.com (S.D.H.); aalnaami@yahoo.com (A.M.A.); ndaghri@ksu.edu.sa (N.A.-D.); 3 Population, Evidence and Technologies, Division of Health Sciences, Warwick Medical School, University of Warwick, Coventry CV2 2 DX, UK; 4 Academic Department of Diabetes, Endocrinology and Metabolism, George Eliot Hospital, Nuneaton CV10 7DJ, UK

**Keywords:** vitamin B12, lipid profile, healthy, Saudi Arabia

## Abstract

An abnormal lipid profile is an independent risk factor for cardiovascular diseases. The relationship between vitamin B12 deficiency and lipid profile is inconclusive, with most studies conducted in unhealthy populations. In this study, we aimed to assess the relationship between serum vitamin B12 levels and lipid profiles in a cross-sectional study that included 341 apparently healthy Saudi women, aged 19–30 years, from different colleges at King Saud University, Saudi Arabia. Sociodemographic, anthropometric, biochemical, and lifestyle data were collected, including diet and physical activity. Serum vitamin B12 deficiency was defined as serum B12 level of <148 pmol/L. The prevalence of vitamin B12 deficiency was approximately 0.6%. Using multivariable linear regression models, serum vitamin B12 levels were found to be inversely associated with total cholesterol (B = −0.26; *p* < 0.001), low-density lipoprotein cholesterol levels (B = −0.30; *p* < 0.001), and triglyceride (B = −0.16; *p* < 0.01) after adjusting for potential confounders, while obesity indices of body mass index, central obesity, and fat percentage showed no association. Therefore, we conclude that low serum vitamin B12 levels are independently associated with abnormal lipid profiles in healthy young Saudi women. Further interventional studies are needed to determine whether improving serum vitamin B12 levels in a healthy population can improve lipid profiles.

## 1. Introduction

Micronutrient deficiencies contribute to the development of many metabolic chronic diseases and are of great importance in global health research, especially in the Middle East [1,2]. Vitamin B12, also known as cobalamin, is a water-soluble vitamin [3] that plays an important role in many cellular functions, such as erythropoiesis, DNA synthesis, and lipid and carbohydrate metabolism [3,4,5]. Vitamin B12 deficiency can develop because of malabsorption, genetic polymorphisms, or low dietary intake [3,6], and it has been associated with health issues ranging from mild fatigue to severe neurological impairment [3,5,7]. According to the World Health Organization (WHO), women of childbearing age are considered a high-risk group for vitamin B12 deficiency [8] and the risk of B12 deficiency may increase by 10%–20% from preconception to early pregnancy [9].

Globally, the prevalence of vitamin B12 deficiency ranges between 2.5% and 40% [6,10,11]. Prevalence among women of childbearing age has been measured at around 12% in the United Kingdom [12] and 34% in Canada [13]. A systematic review showed that during pregnancy, up to 20%–30% of women can be affected by B12 deficiency during all three trimesters, with higher rates among some ethnic groups [14]. The limited number of studies conducted in Arabian countries have suggested that the prevalence of vitamin B12 deficiency ranges from 6% to 30% among diabetes and high-risk populations, respectively [15,16].

Observational studies have shown an inverse association between vitamin B12 intake and metabolic disorders, including body mass index (BMI) [17], insulin resistance [18], type 2 diabetes mellitus (T2DM) [19], adverse lipid profile [20], and cardiovascular diseases (CVDs) [21,22,23]. Animal studies suggest that maternal low B12 levels may be causally linked to adverse lipid profiles in offspring [21]. In addition, vitamin B12 deficiency among pregnant women has been associated with gestational diabetes mellitus [24] and impaired cardiometabolic health of offspring [25]. One suggested mechanism through which vitamin B12 is linked to these disorders is via plasma total homocysteine (tHcy). Both vitamin B12 deficiency and CVDs have been linked to high tHcy concentration [26,27]. tHcy disturbs phospholipid metabolism by affecting the assembly or secretion of very low-density lipoprotein (VLDL), leading to abnormal lipid levels [28]. Other studies have suggested an independent role of vitamin B12, possibly via gene expression involved in lipogenesis [23,29] and inflammation [30]. However, a systematic review of seven prospective cohort studies showed limited and inconclusive results on the role of B12 on CVDs [29], although this could be due to the presence of confounders and the health status of the study samples.

It is important to note that Arabian women of childbearing age have a high risk of metabolic syndrome (29%) [31], prediabetes (40%), and T2DM (35%) [32]. At present, and to the best of our knowledge, no previous studies from the Middle East have assessed vitamin B12 status in relation to lipid profiles in apparently healthy women of childbearing age. Hence, cross-sectional associations of serum vitamin B12 levels and lipid profile indices were investigated using objective measures and detailed dietary data in apparently healthy young women living in Saudi Arabia.

## 2. Materials and Methods

### 2.1. Study Design, Population, and Sample Size

In this study, we included 355 randomly selected women aged between 19 and 30 years with no history of medical issues from different colleges at King Saud University (KSU), Riyadh, Saudi Arabia. Study recruitment was carried out between January and March 2019. Students were invited to participate and were randomly selected from three colleges (sciences, humanities, and medical colleges). Of the 355 women initially selected, 14 individuals were excluded (pregnancy; non-Saudi ethnic group; previous diagnosis of gastrointestinal disorders; anemia; malabsorption; any known chronic conditions such as thyroid disorders, diabetes mellitus, malignancies, and chronic obstructive pulmonary disease; arthritis; and consumption of vitamin B12 supplements or medications with known effects on serum vitamin B12 levels, such as metformin and proton pump inhibitors). The remaining 341 participants provided written informed consent. Among them, 118 (34.6%) were from the humanities college, 112 (32.9%) were from the science college, and 111 (32.6%) were from the medical college. Permission was also obtained for data collection and blood sample stock storage in a biobank at the Chair for Biomarkers of Chronic Diseases (CBCD) laboratories. Ethical approval for this study was obtained from the institutional review board (IRB) of King Khalid University Hospital, Riyadh (IRB number: E-19-3625). The sample size was calculated to identify the relationship between vitamin B12 and lipid profile. At an estimated effect size of 0.20 [33], 95% power, and 95% confidence interval, the required sample size to calculate the correlation between vitamin B12 and triglycerides is 262 subjects. However, to account for a 10% dropout, the minimum sample size was considered 288 subjects. The final sample size in this study was 341.

### 2.2. Biochemical Assessment

Fasting blood was collected after a 10-h overnight fast. A 10 mL sample of venous blood (5 mL serum and 5 mL whole blood) was drawn from the cubital vein. The samples were packaged and transported in a portable refrigerator to the CBCD at KSU. All the samples were aliquoted and stored in a freezer at −80 °C for subsequent analyses.

#### 2.2.1. Lipid Profile and Glucose

Serum total cholesterol (TC), high-density lipoprotein cholesterol (HDL-C), triglyceride (TG), and glucose levels were measured by a colorimetric method using an automated chemistry analyzer (Konelab, ThermoFisher, Finland). The intra- and interassay coefficients of variation (CVs) were TC: 0.7% and 1.5%; HDL-C: 0.6% and 1.2%; TG: 0.9% and 1.8%; and glucose: 0.8% and 2.6%. LDL cholesterol (LDL-C) was calculated using the Friedewald formula [34]. The following thresholds were considered abnormal: TG ≥ 1.7 mmol/L [35], HDL-C < 1.29 mmol/L [36], TC > 5.172 mmol/L, and LDL-C ≥ 3.36 mmol/L [37]. Abnormalities in any one of these parameters are considered indicative of dyslipidemia [38]. Impaired fasting glucose was classified as a glucose level of ≥ 5.6 mmol/L [39].

#### 2.2.2. Vitamin B12

Serum vitamin B12 levels were determined using an electrochemiluminescent immunoassay using a Roche Cobas e411 immunoassay analyzer (Roche Diagnostics, Germany). Vitamin B12 deficiency was defined as a serum vitamin B12 level of <148 pmol/L [40] and insufficiency as <221 pmol/L. The intra- and interassay CVs were 2.9% and 4.1%, respectively.

### 2.3. Dietary Assessment

The Saudi Food and Drug Administration’s food frequency questionnaire was used to measure vitamin B12 intake over the past year [41]. The official Arabic language version of the questionnaire was used to interview individuals. A list of 133 food items was included in the questionnaire and a close-ended approach was used. Nine answer options were provided for each close-ended question, with consumption frequency choices given as follows: never or less than once a month, 1–3 times per month, once a week, 2–4 times per week, 5–6 times per week, once a day, 2–3 times per day, 4–5 times per day, or 6+ times per day. In addition, the questionnaire included open-ended questions, at the end, to gather information about other food items that were not listed. It also included questions regarding the types of cooking fat used, visible fat consumption, and salt and vitamin consumption [41]. The nutritional values of the items were based on the Saudi Food Composition Tables for 1996, McCance and Widdowson’s Composition of Foods Integrated Dataset for 2015, and the 12th edition of the Concise New Zealand Food Composition Tables from 2016 [41,42,43]. In addition, we used another validated questionnaire, “vitamin B12 food questionnaire”, that had been specifically developed to measure vitamin B12 intake from food and beverages [43]. Thus, both questionnaires were integrated to capture the most accurate picture of vitamin B12 intake. The recommended dietary allowance (RDA) for vitamin B12 in adults is 2.4 mcg/day to define adequate daily intake [3].

### 2.4. Clinical Assessment

Anthropometric data were obtained using standard procedures. Weight and height without shoes and heavy clothing were recorded to the nearest 0.2 kg and 0.5 cm, respectively, using an appropriate international standard scale (Digital Pearson Scale, ADAM Equipment Inc., Danbury, CT, USA). BMI (kg/m^2^) was calculated as weight in kilograms divided by height in meters squared. According to the WHO [44], individuals can be categorized into four groups based on BMI: underweight (<18.5 kg/m^2^), normal weight (18.5–24.9 kg/m^2^), overweight (25.0–29.9 kg/m^2^), and obese (≥30 kg/m^2^). Waist and hip circumferences were measured according to WHO procedures. Female participants with a waist circumference of >88 cm were classified as having central obesity, which substantially increased the risk of metabolic complications [45]. Waist-to-hip ratios (WHRs) were obtained by dividing the mean waist circumference by the mean hip circumference. The InBody 770 body composition analyzer (USA, Cerritos, CA, USA) was used for assessing the fat percentage of participants.

### 2.5. Other Risk Factors

All participants were interviewed via a general health history questionnaire that solicited information regarding their sociodemographic background (income and living region) along with family medical history [46]. They were then interviewed using the global physical activity questionnaire (GPAQ).

#### Physical Activity Questionnaire

GPAQ version 2.0 was used to assess physical activity [47], covering several components of physical activity, such as intensity, duration, and frequency, in addition to assessing three domains in which physical activity is performed: occupational physical activity, transport-related physical activity, and physical activity during discretionary or leisure time [48]. This study used the official Arabic version of the GPAQ, which has been used previously for a college-aged Saudi population [47].

### 2.6. Statistical Analysis

Data were analyzed using the SPSS version 23.0 statistical software. The normality of all quantitative variables was tested before performing the analysis. Descriptive statistics (means, standard deviations, medians, quartiles, frequencies, and percentages) were used to quantify the continuous and categorical variables. Data were presented in tertiles of vitamin B12: tertile 1, ≤333.05 pmol/L; tertile 2 ranged from 333.1 to 482.2 pmol/L; and tertile 3, >482.2 pmol/L. Log transformation was used prior to conducting parametric testing. Associations between serum vitamin B12 levels and selected parameters were analyzed using Pearson’s correlation. Multivariate linear and logistic regression models were used to assess the association between serum vitamin B12 levels and the lipid profile per (1 SD = 176.5 pmol/L) of vitamin B12. The models were adjusted for age, BMI, WHR, glucose, income, physical activity, and family history of dyslipidemia and heart disease. *p* < 0.05 was considered to indicate significance.

## 3. Results

### 3.1. Baseline Characteristics by Vitamin B12 Tertiles

The mean age and BMI of participants were 20.7 ± 1.5 years and 23.6 ± 5.2 kg/m^2^, respectively. In total, 14% of participants were overweight, while 14.9% were obese. Moreover, 3.5% of the total participants had central obesity. About 23% and 30.6% of the participants had a family history of dyslipidemia and heart disease, respectively. The demographic characteristics of the participants (e.g., clinical history, income, vitamin B12 intake, physical activity levels) are presented by tertiles of vitamin B12 (Table 1).

Participants with higher serum vitamin B12 levels (tertile 3) had lower levels of TC, LDL-C, and TG levels as well as TC/HDL, TG/HDL, and LDL/HDL ratios compared to participants with a lower serum vitamin B12 level (tertile 1) (Table 1). Macronutrients, energy, and water intake did not show any differences between the three groups (Table S1).

**Figure 1:**
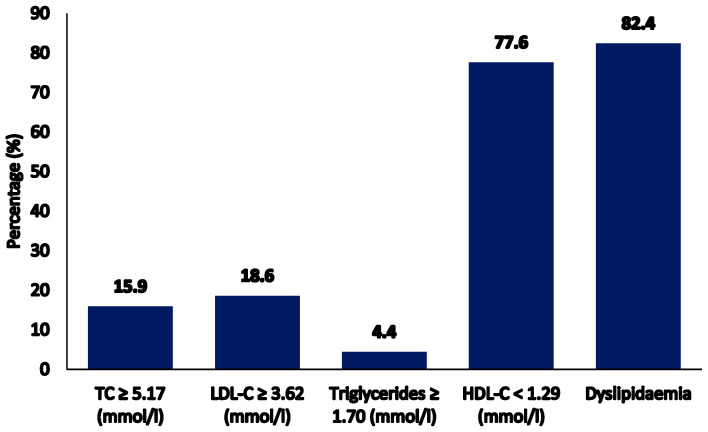
Prevalence of abnormal lipid parameters and dyslipidemia.

The median serum vitamin B12 concentration was 398.9 pmol/L. The prevalence of vitamin B12 deficiency was 0.6% (2/341) and insufficiency was 5.6% (19/341). Of the participants, 15.9% had high TC levels, 18.6% had high LDL-C levels, and 82.4% had dyslipidemia (Figure 1).

### 3.2. Serum Vitamin B12 Levels and Lipid Profile

**Figure 2:**
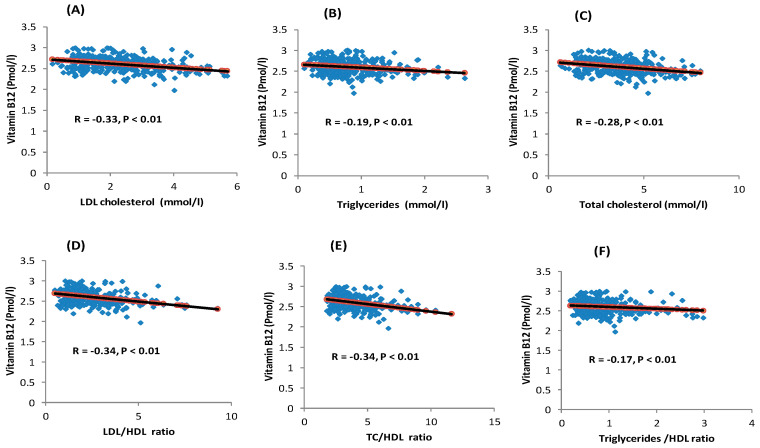
Associations between serum vitamin B12 levels and lipid parameters. (**A**) Association between serum vitamin B12 levels and LDL-C; (**B**) Association between serum vitamin B12 levels and triglycerides; (**C**) Association between serum vitamin B12 levels and total cholesterol; (**D**) Association between serum vitamin B12 levels and LDL/HDL ratio; (**E**) Association between serum vitamin B12 levels and TC/HDL ratio; and (**F**) Association between serum vitamin B12 levels and Triglyceride/HDL ratio. Note: the *p*-value was obtained from the Pearson correlation test.

Pearson’s correlation showed significant inverse associations between serum vitamin B12 levels and the lipid profile parameters, with the exception of HDL-C (Figure 2). Linear regression analyses showed that the serum vitamin B12 level was independently and inversely associated with TC, TG, LDL-C levels, LDL/HDL ratio, TC/HDL ratio, and TG/HDL ratio after adjusting for confounders (Table 2). One SD increase in serum vitamin B12 levels (176.5 pmol/L) reduced TC, TG, and LDL-C by 0.38, 0.07, and 0.34 pmol/L, respectively (Table 2). Multiple logistic regression found that serum vitamin B12 level was inversely related to dyslipidemia but was not significant when adjusted for all confounders. The results were similar when BMI was replaced by either height and WHR, height and fat percentage, or height and central obesity (Table S2).

## 4. Discussion

In a cohort of young women living in Saudi Arabia, we found that serum vitamin B12 levels were inversely associated with the lipid profile (TC, LDL-C, and TG levels), TC/HDL, TG/HDL, and LDL/HDL ratios. The relationship persisted after adjusting for potential confounding factors. The prevalence of dyslipidemia was considered high (around 82%) based on the standard definitions.

To the best of our knowledge, no previous studies have analyzed the prevalence of vitamin B12 deficiency within an apparently healthy young Saudi population. The prevalence of vitamin B12 deficiency found in the current study was 0.6% (insufficiency, 5.5%), which is lower than that reported in other studies in Saudi Arabia and other Arabian countries (i.e., between 6% and 30%) [15,16]. This might be because the high estimated intake of vitamin B12 in our study sample was higher than in others [49]. Nearly 95% of our participants took adequate daily doses of vitamin B12. In addition, some studies from Arabian countries focus on older age groups and patients with diabetes [15,16]. These patients would likely have been on metformin, a commonly used drug for the treatment of diabetes, which is known to cause lower serum vitamin B12 levels [50].

Our findings were consistent with observations in other samples. In a comparative study of patients with T2DM living in the United Kingdom and India, Adaikalakoteswari et al. found that serum vitamin B12 level was independently and inversely associated with levels of TGs and the TC/HDL ratio [33]. Similarly, another cross-sectional study of 300 patients with coronary artery disease reported that serum vitamin B12 level was inversely associated with dyslipidemia as well as TG and VLDL levels and positively associated with HDL-C levels, but was not associated with TC or LDL-C levels [22]. Our study extends this observation to young women in Saudi Arabia. Previous studies have shown that lipid profile ratios are better indicators of CVD than each variable alone [51,52]. Therefore, our observation highlights the importance of understanding the relationship between lipid profile and vitamin B12.

To date, only a few studies have investigated the association of serum vitamin B12 levels with lipid profile in apparently healthy individuals. In a prospective cohort that involved 421 healthy Korean individuals followed up for 12 years, serum vitamin B12 levels were not associated with dyslipidemia or any atherosclerotic events. However, the mean serum vitamin B12 level among Korean individuals was higher than those in other population samples previously studied [53]. Nevertheless, as hypertriglyceridemia is associated with higher rates of gestational diabetes and macrosomia [54], our findings are important as these are observations from young women of child-bearing age. In addition, serum vitamin B12 levels (along with folate) have been linked with adverse pregnancy outcomes [24] and higher insulin resistance in offspring [25].

The mechanism by which vitamin B12 deficiency is associated with the lipid profile may involve elevated plasma tHcy concentrations and affects phospholipid metabolism; this, in turn, causes the high secretion of VLDL, leading to abnormal lipid levels [28]. Another proposed mechanism involves gene expression related to lipogenesis [23,29] and inflammation [30]. The mechanism underlying the relationship with the lipid profile can be further explained by the fact that vitamin B12 acts as a coenzyme in the conversion of methylmalonyl-CoA to succinyl-CoA [55,56]. This reaction is blocked if there are low serum vitamin B12 levels, resulting in methylmalonyl-CoA accumulation, which inhibits the rate-limiting enzyme of fatty acid oxidation [57] and thereby causes lipogenesis.

The current study has the following strengths. First, it involves healthy young women, a group that had not been previously investigated. Second, the extensive data collected in our study on sociodemographic, medical history, dietary information, and physical activity may help identify other important confounding or mediating variables in the association between B12 levels and lipid profiles. However, several limitations are presented in our study. First, it employed a cross-sectional design; therefore, no causal inferences can be made. Second, only the serum vitamin B12 level was used to assess the B12 status. We did not include methylmalonic acid or tHcy levels, indicators of tissue-level B12 deficiency. However, serum vitamin B12 levels have been previously shown to be valid indicators of B12 status in individuals as well as in epidemiological settings [58]. Third, the use of the FFQ and GPAQ questionnaires may have led to recall bias.

In conclusion, we found a high prevalence of dyslipidemia in apparently healthy young women in Saudi Arabia. While it is reassuring to find adequate intake of B12 in this population, our finding of an inverse association between B12 and adverse lipid profile is concerning and warrants further studies. These studies should focus on understanding the mechanisms of the relationship between serum vitamin B12 levels and adverse lipid profiles.

## Figures and Tables

**Table 1:** Characteristics of study participants according to vitamin B12 tertiles.

Parameters	Total	Tertile 1	Tertile 2	Tertile 3	*p*-Value
N	341	114	113	114	
Age (years)	20.7 ± 1.5	20.7 ± 1.6	20.9 ± 1.8	20.7 ± 1.2	0.40
BMI (kg/m^2^)	23.6 ± 5.2	23.9 ± 5.6	23.5 ± 4.7	23.7 ± 5.4	0.85
Waist circumference (cm)	71.1 ± 10.4	72.1 ± 11.0	69.7 ± 9.3	71.4 ± 10.7	0.22
Hip circumference (cm)	99.5 ± 11.7	100.1 ± 10.7	98.7 ± 13.1	99.5 ± 11.1	0.66
Waist/hip ratio	0.72 ± 0.06	0.72 ± 0.07	0.70 ± 0.05 ^A^	0.72 ± 0.06	0.03
Fat (%)	36.9 ± 8.2	37.1 ± 8.1	36.8 ± 8.0	37.3 ± 8.1	0.91
Family history of dyslipidemia	8 (2.3)	2 (1.8)	5 (4.4)	1 (0.9)	0.19
Family history of hyperlipidemia	129 (37.8)	42 (36.8)	50 (44.2)	37 (32.5)	0.19
Family history of heart disease	104 (30.6)	39 (34.2)	35 (31.0)	30 (26.5)	0.55
Income level (<10,000 SAR)	70 (20.5)	26 (22.8)	24 (21.2)	20 (17.5)	0.60
Vitamin B12 intake (mcg/day)	6.9 (4.4–10.8)	6.3 (3.5–10.1)	6.8 (4.9–9.9)	8.3 (4.8–11.6) ^A^	0.02
Adequate vitamin B12 (≥2.4 mcg/day)	323 (94.7)	102 (89.5)	109 (96.5)	112 (98.2) ^A^	0.01
Median B12 levels	398.9 (305.8–534.6)	269.1 (243.0–305.8)	398.9 (361.0–448.3) ^A^	596.6 (534.6–683.7) ^AB^	<0.001
GPAQ score (MET-minute/week) ^#^	504.0 (160.0–1240.0)	600.0 (200.0–1620.0)	560.0 (160.0–1200.0)	400.0 (180.0–940.0)	0.18
**Biochemical characteristics**					
Fasting glucose (mmol/L)	4.6 ± 1.0	4.6 ± 1.0	4.6 ± 1.1	4.7 ± 0.9	0.77
TC (mmol/L)	3.8 ± 1.4	4.3 ± 1.6	3.8 ± 1.4 ^A^	3.3 ± 1.2 ^A^	<0.001
HDL-C (mmol/L)	1.0 ± 0.4	1.0 ± 0.3	1.0 ± 0.4	1.1 ± 0.4	0.34
LDL-Cl (mmol/L)	2.4 ± 1.2	2.8 ± 1.3	2.3 ± 1.0 ^A^	2.0 ± 0.9 ^A^	<0.001
TG (mmol/L)	0.8 ± 0.4	0.9 ± 0.5	0.7 ± 0.4 ^A^	0.7 ± 0.4 ^A^	<0.001
TC/HDL ratio	3.9 ± 1.6	4.6 ± 2.0	3.8 ± 1.4 ^A^	3.3 ± 1.0 ^A^	<0.001
TG/HDL ratio	0.9 ± 0.6	1.0 ± 0.6	0.8 ± 0.8	0.7 ± 0.4 ^A^	<0.01
LDL-C/HDL-C	2.4 ± 1.4	3.0 ± 1.7 ^A^	2.4 ± 1.2 ^A^	2.0 ± 0.9	<0.001

Note: data presented as mean ± SD for normal variables and median (IQR) for non-normal variables; ^#^ indicates non-normal variables; superscript A and B indicates significance from tertile 1 and tertile 2, respectively; *p*-values were obtained from ANOVA and Kruskal–Wallis H tests for normal and non-normal variables, respectively; significance is set at *p* < 0.05. GPAQ; global physical activity questionnaire.

**Table 2:** Associations between vitamin B12 and lipid profile per 1 SD vitamin B12; *n* = 341.

Parameters	Model 1			Model 2		
	B ± SE/OR	B (S)/95%CI	*p*-Value	B ± SE	B (S)/95%CI	*p*-Value
Total cholesterol (mmol/L)	−0.39 ± 0.08	−0.27	<0.0001	−0.38 ± 0.07	−0.26	<0.0001
Triglycerides (mmol/L)	−0.07 ± 0.02	−0.17	<0.01	−0.07 ± 0.02	−0.16	<0.01
LDL-C (mmol/L)	−0.35 ± 0.06	−0.30	<0.0001	−0.34 ± 0.06	−0.30	<0.0001
HDL-C (mmol/L)	0.01 ± 0.02	0.04	0.51	0.01 ± 0.02	0.04	0.48
LDL/HDL ratio	−0.41 ± 0.07	−0.30	<0.0001	−0.41 ± 0.07	−0.30	<0.0001
TC/HDL ratio	−0.48 ± 0.09	−0.30	<0.0001	−0.47 ± 0.09	−0.29	<0.0001
Triglyceride/HDL ratio	−0.09 ± 0.04	−0.14	0.02	−0.08 ± 0.04	−0.13	0.02
Dyslipidemia *	0.75	0.56–1.00	0.05	0.74	0.55–1.01	0.06

Note: data presented as B ± SE and odds ratio for continuous and categorical variables were obtained from linear and logistic regression models, respectively; the lipid profile was the dependent variable. Model 1 adjusted for age, BMI, physical activity, income, family history of hyperlipidemia, and heart disease; Model 2 adjusted for Model 1 and glucose. *p* < 0.05 was considered significant. * indicates categorical variables; *p* < 0.05 is considered significant.

## Supplementary Materials

The following are available online at https://www.mdpi.com/2072-6643/12/8/2395/s1, Table S1: Dietary intake of study participants by vitamin B12 tertiles, Table S2: Associations between vitamin B12 and lipid profile.

## References

[1] Zalaket J., Wehbe T., Jaoude E.A. (2018). Vitamin B12 Deficiency in Diabetic Subjects Taking Metformin: A Cross Sectional Study in a Lebanese Cohort. J. Nutr. Intermed. Metab..

[2] Al-Daghri N.M., Al-Attas O.S., Alokail M.S., Alkharfy K.M., Yakout S.M., Aljohani N.J., Alfawaz H.A., Al-Ajlan A.S.M., Sheshah E.S., Al-Yousef M. (2014). Lower Vitamin D Status is More Common Among Saudi Adults with Diabetes Mellitus Type 1 Than in Non-diabetics. BMC Public Health.

[3] Institute of Medicine (US) Standing Committee on the Scientific Evaluation of Dietary Reference Intakes (1998). Dietary Reference Intakes for Thiamin, Riboflavin, Niacin, Vitamin b6, Folate, Vitamin b12, Pantothenic Acid, Biotin, and Choline.

[4] Saravanan P., Yajnik C.S. (2010). Role of Maternal Vitamin B12 on the Metabolic Health of the Offspring: A Contributor to the Diabetes Epidemic?. Br. J. Diabetes Vasc. Dis..

[5] Rolfes S.R., Pinna K., Whitney E. (2012). Understanding Normal and Clinical Nutrition.

[6] Palacios G., Sola R., Barrios L., Pietrzik K., Castillo M.J., González-Gross M. (2013). Algorithm for the Early Diagnosis of Vitamin B12 Deficiency in Elderly People. Nutr. Hosp..

[7] Mccombe P.A., Mcleod J.G. (1984). The Peripheral Neuropathy of Vitamin B12 Deficiency. J. Neurol. Sci..

[8] de Benoist B. (2008). Conclusions of a WHO Technical Consultation on Folate and Vitamin B12 Deficiencies. Food Nutr. Bull..

[9] Murphy M.M., Molloy A.M., Ueland P.M., Fernandez-Ballart J.D., Schneede J., Arija V., Scott J.M. (2007). Longitudinal Study of the Effect of Pregnancy on Maternal and Fetal Cobalamin Status in Healthy Women and Their Offspring. J. Nutr..

[10] Bailey R.L., Carmel R., Green R., Pfeiffer C.M., Cogswell M.E., Osterloh J.D., Sempos C.T., Yetley E.A. (2011). Monitoring of Vitamin B-12 Nutritional Status in the United States by Using Plasma Methylmalonic Acid and Serum Vitamin B-12. Am. J. Clin. Nutr..

[11] Green R., Allen L.H., Bjørke-Monsen A.-L., Brito A., Guéant J.-L., Miller J.W., Molloy A.M., Nexo E., Stabler S., Toh B.-H. (2017). Vitamin B12 Deficiency. Nat. Rev. Dis. Primers.

[12] Sukumar N., Adaikalakoteswari A., Venkataraman H., Maheswaran H., Saravana P. (2016). Vitamin B12 Status in Women of Childbearing Age in the UK and Its Relationship with National Nutrient Intake Guidelines: Results From Two National Diet and Nutrition Surveys. BMJ Open.

[13] Quay T.A., Schroder T.H., Jeruszka-Bielak M., Li W., Devlin A.M., Barr S.I., Lamers Y. (2015). High Prevalence of Suboptimal Vitamin B12 Status in Young Adult Women of South Asian and European Ethnicity. Appl. Physiol. Nutr. Metab..

[14] Sukumar N., Rafnsson S.B., Kandala N.B., Bhopal R., Yajnik C.S., Saravanan P. (2016). Prevalence of Vitamin B-12 Insufficiency During Pregnancy and Its Effect on Offspring Birth Weight: A Systematic Review and Meta-Analysis. Am. J. Clin. Nutr..

[15] El-Khateeb M., Khader Y., Batieha A., Jaddou H., Hyassat D., Belbisi A., Ajlouni K. (2014). Vitamin B12 Deficiency in Jordan: A Population-Based Study. Ann. Nutr. Metab..

[16] Alharbi T.J., Tourkmani A.M., Abdelhay O., Alkhashan H.I., Al-Asmari A.K., Bin Rsheed A.M., Abuhaimed S.N., Mohammed N., AlRasheed A.N., AlHarbi N.G. (2018). The Association of Metformin Use with Vitamin B12 Deficiency and Peripheral Neuropathy in Saudi Individuals With Type 2 Diabetes Mellitus. PLoS ONE.

[17] Sun Y., Sun M., Liu B., Du Y., Rong S., Xu G., Snetselaar L.G., Bao W. (2019). Inverse Association Between Serum Vitamin B12 Concentration and Obesity Among Adults in the United States. Front. Endocrinol..

[18] Knight B.A., Shields B.M., Brook A., Hill A., Bhat D.S., Hattersley A.T., Yajnik C.S. (2015). Lower Circulating B12 is Associated with Higher Obesity and Insulin Resistance During Pregnancy in a Non-Diabetic White British Population. PLoS ONE.

[19] Krishnaveni G.V., Hill J.C., Veena S.R., Bhat D.S., Wills A.K., Karat C.L., Yajnik C.S., Fall C.H. (2009). Low Plasma Vitamin B12 in Pregnancy is Associated with Gestational ‘Diabesity’ and Later Diabetes. Diabetologia.

[20] Saraswathy K.N., Joshi S., Yadav S., Garg P.R. (2018). Metabolic Distress in Lipid and One Carbon Metabolic Pathway Through Low Vitamin B-12: A Population Based Study from North India. Lipids Health Dis..

[21] Boachie J., Adaikalakoteswari A., Samavat J., Saravanan P. (2020). Low Vitamin B12 and Lipid Metabolism: Evidence from Pre-Clinical and Clinical Studies. Nutrients.

[22] Mahalle N., Kulkarni M.V., Garg M.K., Naik S.S. (2013). Vitamin B12 Deficiency and Hyperhomocysteinemia as Correlates of Cardiovascular Risk Factors in Indian Subjects with Coronary Artery Disease. J. Cardiolol..

[23] Adaikalakoteswari A., Finer S., Voyias P.D., McCarthy C.M., Vatish M., Moore J., Smart-Halajko M., Bawazeer N., Al-Daghri N.M., McTernan P.G. (2015). Vitamin B12 Insufficiency Induces Cholesterol Biosynthesis by Limiting S-Adenosylmethionine and Modulating the Methylation of SREBF1 and LDLR Genes. Clin. Epigenetics.

[24] Sukumar N., Venkataraman H., Wilson S., Goljan I., Selvamoni S., Patel V., Saravanan P. (2016). Vitamin B12 Status Among Pregnant Women in the UK and Its Association with Obesity and Gestational Diabetes. Nutrients.

[25] Stewart C.P., Christian P., Schulze K.J., Arguello M., LeClerq S.C., Khatry S.K., West K.P. (2011). Low Maternal Vitamin B-12 Status is Associated with Offspring Insulin Resistance Regardless of Antenatal Micronutrient Supplementation in Rural Nepal. J. Nutr..

[26] Bailey L.B., Stover P.J., McNulty H., Fenech M.F., Gregory J.F., Mills J.L., Pfeiffer C.M., Fazili Z., Zhang M., Ueland P.M. (2015). Biomarkers of Nutrition for Development-Folate Review. J. Nutr..

[27] Keser I., Ilich J.Z., Vrkić N., Giljević Z., Colić Barić I. (2013). Folic Acid and Vitamin B(12) Supplementation Lowers Plasma Homocysteine but has no Effect on Serum Bone Turnover Markers in Elderly Women: A Randomized, Double-Blind, Placebo-Controlled Trial. Nutr. Res..

[28] Obeid R., Herrmann W. (2009). Homocysteine and Lipids: S-Adenosyl Methionine as a Key Intermediate. FEBS Lett..

[29] Rafnsson S.B., Saravanan P., Bhopal R.S., Yajnik C.S. (2011). Is a Low Blood Level of Vitamin B12 a Cardiovascular and Diabetes Risk Factor? A Systematic Review of Cohort Studies. Eur. J. Nutr..

[30] Kumar K.A., Lalitha A., Pavithra D., Padmavathi I.J., Ganeshan M., Rao K.R., Venu L., Balakrishna N., Shanker N.H., Reddy S.U. (2013). Maternal Dietary Folate and/or Vitamin B12 Restrictions Alter Body Composition (Adiposity) and Lipid Metabolism in Wistar Rat Offspring. J. Nutr. Biochem..

[31] Al-Rubeaan K., Bawazeer N., Al Farsi Y., Youssef A.M., Al-Yahya A.A., AlQumaidi H., Al-Malki B.M., Naji K.A., Al-Shehri K., Al Rumaih F.I. (2018). Prevalence of Metabolic Syndrome in Saudi Arabia—A Cross Sectional Study. BMC Endocr. Disord..

[32] Al-Rifai R.H., Majeed M.I., Tariq M.R., Irfan A. (2019). Type 2 Diabetes and Pre-Diabetes Mellitus: A Systematic Review and Meta-Analysis of Prevalence Studies in Women of Childbearing Age in the Middle East and North Africa, 2000-2018. Syst. Rev..

[33] Adaikalakoteswari A., Jayashri R., Sukumar N., Venkataraman H., Pradeepa R., Gokulakrishnan K., Anjana R.M., McTernan P.G., Tripathi G., Patel V. (2014). Vitamin B12 Deficiency is Associated with Adverse Lipid Profile in Europeans and Indians With Type 2 Diabetes. Cardiovasc. Diabetol..

[34] Friedewald W.T., Levy R.I., Fredrickson D.S. (1972). Estimation of the Concentration of Low-Density Lipoprotein Cholesterol in Plasma, Without Use of the Preparative Ultracentrifuge. Clin. Chem..

[35] Grundy S.M., Cleeman J.I., Daniels S.R., Donato K.A., Eckel R.H., Franklin B.A., Gordon D.J., Krauss R.M., Savage P.J., Smith S.C. (2005). Diagnosis and Management of the Metabolic Syndrome: An American Heart Association/National Heart, Lung, and Blood Institute Scientific Statement. Circulation.

[36] Saely C.H., Koch L., Schmid F., Marte T., Aczel S., Langer P., Hoefle G., Drexel H. (2006). Adult Treatment Panel III 2001 but not International Diabetes Federation 2005 Criteria of the Metabolic Syndrome Predict Clinical Cardiovascular Events in Subjects Who Underwent Coronary Angiography. Diabetes Care.

[37] Jellinger P.S., Handelsman Y., Rosenblit P.D., Bloomgarden Z.T., Fonseca V.A., Garber A.J., Grunberger G., Guerin C.K., Bell D.S.H., Mechanick J.I. (2017). American Association of Clinical Endocrinologists and American College of Endocrinology Guidelines for Management of Dyslipidemia and Prevention of Cardiovascular Disease. Endocr. Pract..

[38] Stone N.J., Robinson J.J., Lichtenstein A.H., Merz C.N.B., Blum C.B., Eckel R.H., Goldberg A.C., Gordon D., Levy D., Lloyd-Jones D.M. (2014). 2013 ACC/AHA Guideline on the Treatment of Blood Cholesterol to Reduce Atherosclerotic Cardiovascular Risk in Adults: A Report of the American College of Cardiology/American Heart Association Task Force on Practice Guidelines. J. Am. Coll. Cardiol..

[39] Genuth S., Alberti K.G., Bennett P., Buse J., Defronzo R., Kahn R., Kitzmiller J., Knowler W.C., Lebovitz H., Lernmark A. (2003). Follow-Up Report on the Diagnosis of Diabetes Mellitus. Diabetes Care.

[40] Hunt A., Harrington D., Robinson S. (2014). Vitamin B12 Deficiency. BMJ.

[41] Alkhalaf M., Edwards C., Combet E. (2015). Validation of a Food Frequency Questionnaire Specific for Salt Intake in Saudi Arabian Adults Using Urinary Biomarker and Repeated Multiple Pass 24-Hour Dietary Recall. Proc. Nutr. Soc..

[42] Roe M., Pinchen H., Church S., Finglas P. (2015). McCance and Widdowson’s The Composition of Foods Seventh Summary Edition and Updated Composition of Foods Integrated Dataset. Nutr. Bull..

[43] Mearns G.J., Rush E.C. (2017). Screening for Inadequate Dietary Vitamin B-12 Intake in South Asian Women Using a Nutrient-Specific, Semi-Quantitative Food Frequency Questionnaire. Asia Pac. J. Clin. Nutr..

[44] Bray G.A. (1999). Clinical Evaluation of the Obese Patient. Best Pract. Res. Clin. Endocrinol. Metab..

[45] World Health Organizaion (2018). Obesity and Overweight. http://www.who.int/news-room/fact-sheets/detail/obesity-and-overweight.

[46] Al-Musharaf S., Fouda M.A., Turkestani I.Z., Al-Ajlan A., Sabico S., Alnaami A.M., Wani K., Hussain S.D., Alraqebah B., Al-Serehi A. (2018). Vitamin D Deficiency Prevalence and Predictors in Early Pregnancy Among Arab Women. Nutrients.

[47] Alkahtani S.A. (2016). Convergent Validity: Agreement Between Accelerometry and the Global Physical Activity Questionnaire in College-Age Saudi Men. BMC Res. Notes.

[48] World Health Organization Global Physical Activity Questionnaire (GPAQ) Analysis Guide: World Health Organization. http://www.who.int/chp/steps/resources/GPAQ_Analysis_Guide.pdf.

[49] Vogiatzoglou A., Smith A.D., Nurk E., Berstad P., Drevon C.A., Ueland P.M., Vollset S.E., Tell G.S., Refsum H. (2009). Dietary Sources of Vitamin B-12 and Their Association with Plasma Vitamin B-12 Concentrations in the General Population: The Hordaland Homocysteine Study. Am. J. Clin. Nutr..

[50] Aroda V.R., Edelstein S.L., Goldberg R.B., Knowler W.C., Marcovina S.M., Orchard T.J., Bray G.A., Schade D.S., Temprosa M.G., White N.H. (2016). Long-term Metformin Use and Vitamin B12 Deficiency in the Diabetes Prevention Program Outcomes Study. J. Clin. Endocrinol. Metab..

[51] Ingelsson E., Schaefer E.J., Contois J.H., McNamara J.R., Sullivan L., Keyes M.J., Pencina M.J., Schoonmaker C., Wilson P.W., D’Agostino R.B. (2007). Clinical Utility of Different Lipid Measures for Prediction of Coronary Heart Disease in Men and Women. JAMA.

[52] Russo G.T., Giandalia A., Romeo E.L., Marotta M., Alibrandi A., De Francesco C., Horvath K.V., Asztalos B.F., Cucinotta D. (2014). Lipid and non-lipid cardiovascular risk factors in postmenopausal type 2 diabetic women with and without coronary heart disease. J. Endocrinol. Investig..

[53] Kim H.-N., Eun Y.-M., Song S.-W. (2019). Serum Folate and Vitamin B 12 Levels are not Associated with the Incidence Risk of Atherosclerotic Events Over 12 Years: The Korean Genome and Epidemiology Study. Nutr. Res..

[54] Gorban de Lapertosa S., Alvariñas J., Elgart J.F., Salzberg S., Gagliardino J.J., EduGest Group (2020). The Triad Macrosomia, Obesity, and Hypertriglyceridemia in Gestational Diabetes. Diabetes Metab. Res. Rev..

[55] Strain J.J., Dowey L., Ward M., Pentieva K., McNulty H. (2004). B-Vitamins, Homocysteine Metabolism and CVD. Proc. Nutr. Soc..

[56] Rosenberg I.H. (2008). Metabolic Programming of Offspring by Vitamin B12/folate Imbalance During Pregnancy. Diabetologia.

[57] Brindle N.P., Zammit V.A., Pogson C.I. (1985). Regulation of Carnitine Palmitoyltransferase Activity by malonyl-CoA in Mitochondria from Sheep Liver, a Tissue With a Low Capacity for Fatty Acid Synthesis. Biochem. J..

[58] Carmel R. (2011). Biomarkers of Cobalamin (Vitamin B-12) Status in the Epidemiologic Setting: A Critical Overview of Context, Applications, and Performance Characteristics of Cobalamin, Methylmalonic Acid, and Holotranscobalamin II. Am. J. Clin. Nutr..

