# Supplementary material for: Low Serum Vitamin B12 Levels Are Associated with Adverse Lipid Profiles in Apparently Healthy Young Saudi Women
Source: Nutrients. 2020 Aug 10;12(8):2395. doi: 10.3390/nu12082395 (PMC7468727; doi:10.3390/nu12082395)
Supplement: Supplementary file 1 [file nutrients-12-02395-s001.pdf]

Supplementary Table S1: Dietary intake of study participants by vitamin B12 tertiles

Parameters	Total	Tertile 1 (≤333.05 pmol/l)	Tertile 2 (333.1–482.2)	Tertile 3 (>482.2)	P value
N	341	113	114	114	
Energy (kcal/day)#	2919 (2133–3810)	2910 (2099–3789)	2990 (2188–3890)	2827 (2027–3690)	0.861
Fat (gm/day)#	125 (83–179)	127 (89–165)	125 (82–199)	124.0 (81.3–173.3)	0.956
Protein (gm/day)#	105 (78.0–142)	100.3 (79.7–140.0)	108.9 (79.3–138.7)	113.0 (78.0–146.9)	0.622
Carbohydrate (gm/day)#	368 (268–479)	370 (282–504)	376 (271–482)	365 (250–469)	0.738
Fiber (gm/day)#	30 (20–42)	31 (20–42)	29.5 (19.0–43)	29.6 (21.4–41.0)	0.689
Water (ml/day)#	1698 (1259–2217)	1661(1270–2209)	1751.5 (1281–2208)	1681.2 (1231–2330)	0.952

Note: Data presented as median (IQR); # indicates non-normal variables; Superscript A and B indicates significance from Tertile 1 and Tertile 2 respectively; P-values are obtained from Kruskal–Wallis H test .* indicates P-values <0.05.

Supplementary Table S2: Associations between vitamin B12 and lipid profile

Parameters	Model3a			Model 3b			Model 3c		
	B ± SE	B (S)	P-value	B ± SE	B (S)	P-value	B ± SE	B (S)	P value
Total cholesterol (mmol/l)	-0.38 ± 0.07	-0.26	<0.0001	-0.38 ± 0.07	-0.26	<0.0001	-0.38 ± 0.07	-0.26	<0.0001
Triglycerides (mmol/l)	-0.07 ± 0.02	-0.16	0.003	-0.07 ± 0.02	-0.16	0.003	-0.07 ± 0.02	-0.16	0.003
LDL-C (mmol/l)	-0.34 ± 0.06	-0.30	<0.0001	-0.34 ± 0.06	-0.30	<0.0001	-0.34 ± 0.06	-0.30	<0.0001
HDL-C (mmol/l)	0.01 ± 0.02	0.03	0.510	0.01 ± 0.02	0.04	0.472	0.01 ± 0.02	0.03	0.513
LDL-HDL ratio	-0.41 ± 0.07	-0.30	<0.0001	-0.41 ± 0.07	-0.30	<0.0001	-0.41 ± 0.08	-0.30	<0.0001
TC-HDL ratio	-0.48 ± 0.09	-0.29	<0.0001	-0.48 ± 0.09	-0.30	<0.0001	-0.47 ± 0.09	-0.29	<0.0001
Triglyceride-HDL ratio	-0.08 ± 0.04	-0.13	0.022	-0.08 ± 0.04	-0.13	0.021	-0.08 ± 0.04	-0.13	0.024
Dyslipidaemia*	0.75	0.55–1.01	0.058	0.75	0.55–1.01	0.057	0.74	0.55–1.00	0.052

Note: Data presented as B ± SE and Odds ratio for continuous and categorical variables were obtained from linear and logistic regression model respectively; lipid profile was the dependent variable. Model 3a adjusted for age, height, WHR, physical activity, income, family history of hyperlipidaemia and heart disease; Model 3b adjusted for age, height, fat%, physical activity, income, family history of hyperlipidaemia and heart

disease; Model 3c for age, height, central obesity, physical activity, income, family history of hyperlipidaemia and heart disease * Indicates categorical variables; $P<0.05$ is considered
